# A user task design notation for improved software design

**DOI:** 10.7717/peerj-cs.503

**Published:** 2021-05-24

**Authors:** Eda Ozcan, Damla Topalli, Gul Tokdemir, Nergiz Ercil Cagiltay

**Affiliations:** 1 Vakifbank, Ankara, Turkey; 2 Computer Engineering Department, Atilim University, Ankara, Turkey; 3 Computer Engineering Department, Cankaya University, Ankara, Turkey; 4 Software Engineering Department, Atilim University, Ankara, Turkey

**Keywords:** UML-Activity diagram, Software quality, Software design, Player task notation, Defect detection performance

## Abstract

System design is recognized as one of the most critical components of a software system that bridges system requirements and coding. System design also has a significant impact on testing and maintenance activities, and on further improvements during the lifespan of the software system. Software design should reflect all necessary components of the requirements in a clear and understandable manner by all stakeholders of the software system. To distinguish system elements, separation of concerns in software design is suggested. In this respect, identification of the user tasks, i.e., the tasks that need to be performed by the user, is not currently reflected explicitly in system design documents. Our main assumption in this study is that software quality can be improved significantly by clearly identifying the user tasks from those that need to be performed by the computer system itself. Additionally, what we propose has the potential to better reflect the user requirements and main objectives of the system on the software design and thereby to improve software quality. The main aim of this study is to introduce a novel notation for software developers in the frame of UML Activity Diagram (UML-AD) that enables designers to identify the user tasks and define them separately from the system tasks. For this purpose, an extension of UML-AD, named UML-ADE (UML-Activity Diagram Extended) was proposed. Afterwards, it was implemented in a serious game case for which the specification of user tasks is extremely important. Finally, its effectiveness was analyzed and compared to UML-AD experimentally with 72 participants. The defect detection performance of the participants on both diagrams with two real-life serious game scenarios was evaluated. Results show a higher level of understandability for those using UML-ADE, which in turn may indicate a better design and higher software quality. The results encourage researchers to develop specific design representations dedicated to task design to improve system quality and to conduct further evaluations of the impact of these design on each of the above mentioned potential benefits for the software systems.

## Introduction

Over the last decade, software engineers have recognized the importance of system design that represents both functional and non-functional system requirements. Information related to software design is one of the critical determiners of the lifespan of a system (Booch et al., 2008). System design acts like a bridge between requirements and coding and hence, the improvements in the design processes may offer several advantages in terms of effectiveness of system development activities such as traceability, complexity, and evolution (Rumbaugh et al., 1991). Clear design representation supports the enumeration and description of programmers’ concerns about the system. Here, ‘concerns’ refers to the domain elements in a software system that can be recognized based on features, aspects, roles, and viewpoints (Kiczales et al., 1997; Reid Turner et al., 1999). Separation of concerns is a concept that refers to modularizing the system elements that can be identified based on purpose, goal, or scope (Daga, De Cesare & Lycett, 2006). Focusing designers’ interest on a single concern supports the handling of complexity in a software system. Separation of concerns simplifies comprehension by decoupling the complex system. Hence, the various concerns need to be handled in ‘isolation’ of each other (Goderis, 2008). The technique of separating concerns provide a deconstruction that can separate overlapping concerns, which supports reuse and perception (Daga, De Cesare & Lycett, 2006). One of the concerns that needs to be identified during the system requirements studies, and to be reflected in the software design, is the user tasks. The identification of user tasks in various software systems is critical. For instance, it has been reported that in agile user-centered design process, the specialists encounter difficulty in building links between user tasks and application features (Lee et al., 2010). Measuring human performance and the success and failure scenarios for a specific task can also be considered a critical concern for serious-game design process. That is why representing the player-task design for serious games is important to better motivate users and provide appropriate enforcement (Lim & Reeves, 2010). It has been reported that in use case diagrams it is not possible to represent functions that are not initiated by a user like notifications or events given by a system (Iqbal et al., 2020). Similarly, system related behavior or events cannot be represented in UML-AD diagrams. Therefore, there is a need for a specific notation in system initiated events. In SG, user interacts with the system to play the game and, system and user behaviours need to be distinguished during design phase. This is not possible with UML-AD. However, in the literature there are few studies such as (Lee et al., 2010; Memmel, Gundelsweiler & Reiterer, 2007) that consider the identification of user tasks. In addition, the current software design tools’ ability to identify and represent the user tasks in the software design is limited. Accordingly, in this study, the effectiveness of explicitly identifying and representing user task in the software design document is researched. In other words, we intend to analyze participants’ defect detection performance on software design diagrams that are prepared using UML-ADE. By identifying the user tasks explicitly, the intention is to increase the level of understandability, which may also improve the rate of defect detection at a very early stage. Finally, by providing more explicit design documentation, it is expected that software quality will be improved and that development costs will be lowered.

## Background

Detecting the defects as early as possible during the software life cycle is an important and critical task. According to Boehm & Basili (2005), 40–50% of the development effort is spent on fixing defects that could have been detected (and presumably fixed) earlier in the development process (Boehm & Basili, 2005). Studies also show that it is possible to increase the rates of error detection in the early stages of the software life-cycle by using model-based approaches (Alur & Chandrashekharapuram, 2007; Kof, 2007; Kof et al., 2010). As it offers a clear view of multiple aspects of system design, the conceptual model of software systems is important. The conceptual model also acts as a communication tool for the stakeholders of the development process.

### Need for the representation of system tasks in the conceptual model

The main assumption of this study is that, by explicitly designing system requirements in the conceptual models, their levels of understandability and the error detection processes could be improved. For instance, for some specific software like serious games, there is a need for representing the system tasks and user tasks separately and explicitly. These so-called serious games are designed not only for pure entertainment but also for serious purposes such as training, scientific research, or advertising (Michael & Chen, 2005). In contrast to the studies that show advantages of serious games, others report several challenges of them (Bellotti, Berta & De Gloria, 2010; Fernández-Manjón et al., 2015). As there are limited implementations of serious games in the educational environments, their socio-cultural, educational, and technological challenges need to be analyzed deeply (Fernández-Manjón et al., 2015). To face these challenges, the earlier studies also suggest new design approaches and tools that considered a variety of user types for more effective serious-game design (Bellotti, Berta & De Gloria, 2010). As earlier studies report, serious games should provide testing and progress tracking to evaluate how much a user has learned from playing and architectures that reflect elements of personalization and experience specific to the user (Michael & Chen, 2005; Bellotti, Berta & De Gloria, 2010). It is also reported that participants, rules and procedures, and success and failure are key components of any purposeful human activity (Abt, 1987). Additionally, besides the main components of a game like story and art, a serious game also involves pedagogy that supports elements of fun (Zyda, 2005). Hence, a serious game can be considered a computer-based challenge that aims to improve training through entertainment (Zyda, 2005). All these constraints address the identification of player tasks explicitly, which may have an impact on the assessment of the user performance, and on the feedback mechanism by the software, which in turn affects the user experience and user interfaces of the software product. Serious games are non-simple systems and hence their design requires special attention (Barral et al., 2020).

### UML-AD as a conceptual design tool for serious games

In order to address these challenges of the serious games, several studies explored using Unified Modeling Language (UML). For instance, for generally modeling the expert reasoning in serious games, (Barral et al., 2020) have proposed an UML profile library. As UML is a very well-known and highly popular modeling language, several researches explored its extensions to adopt it into different contexts or purposes. Many UML-based representations have been studied which address specific requirements of various domains. AODML (Vyas, Vishwakarma & Jha, 2017) is proposed as a modeling language for aspect-oriented programming domain that provides capability of specification of aspects in the design and implementation phases. Secure-UML (Lodderstedt, Basin & Doser, 2002) incorporates the access control infrastructures in the design. For medical image processing, StarUML is proposed that captures annotations of medical images Ayadi, Bouslimi & Akaichi, 2013). IoTsec is another example that includes a notation for model security in IoT systems (Robles-Ramirez, Escamilla-Ambrosio & Tryfonas, 2017). Similarly, Agent UML enables description of agent behavior (Bauer, Müller & Odell, 2001). Additionally, UML is extended to include some other artifacts such as model checking (Shu, Wang & Wang, 2017), model merging (Farias et al., 2019). Currently, UML is the most popular conceptual modeling language (Mohagheghi, Dehlen & Neple, 2009; Torchiano et al., 2013) and offers a communication platform for software engineers to define, build, visualize a software system (Booch, Rumbaugh & Jacobson, 2005). It provides various diagrams for static and dynamic behaviors of a software system. As a standardized modeling language used for software design tasks, UML is a powerful tool that benefits not only software developers but also other stakeholders (Cooper, Nasr & Longstreet, 2014). In order to better design the games in general and game theory modeling (Noreen & Saxena, 2017) in particular, and in order to better validate and review system requirements inspection, many researchers reported benefits of the Unified Modelling Language Activity Diagrams (UML-AD) (Dobing & Parsons, 2006; Muñoz, Mazón & Trujillo, 2010; Bolloju & Sun, 2012). UML-AD describes the dynamic characteristics of a system. It represents system behavior through the flow between system activities. In addition, UML-AD is a popular and powerful design tool to represent task flow design in serious game environments (Ismailović et al., 2012; Buendía-García et al., 2013; De Lope & Medina-Medina, 2016; Callaghan et al., 2018).

### Representation of system tasks in UML-AD

To identify and to separate the system tasks from the user tasks in UML-AD representations, swimlanes can be used. In the UML-AD, these tasks are all represented by the same action notation. As reported in earlier studies, some effort is still required to better implement this notation into the software development life cycle (France et al., 1998). Accordingly, in this study, an extension to the UML-AD representation (named as UML-ADE notation) has been proposed and its impact on the understandability of the software design is evaluated experimentally for a serious game case.

## Materials & Methods

**Figure 1:**
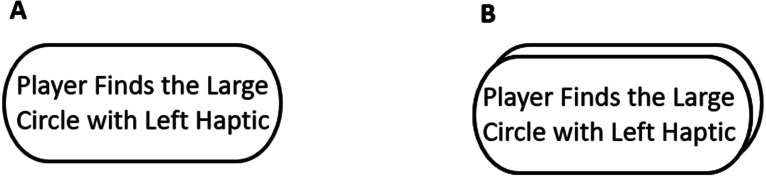
Player task notations (A) UML-AD. (B) UML-ADE. Figure shows the notations used for computer and user tasks.

Based on the objectives set forth in this paper, a graphical notation for user tasks is proposed to distinguish user and system tasks. Accordingly, double-circled action representation for the UML-ADE is proposed. In other words, in the UML-ADE model, the standard UML-AD action notation is used to represent the system tasks, while double circled action notation is used to represent the user tasks (Fig. 1).

To evaluate the proposed UML-ADE representation, two scenarios for a game-like surgical simulation environment were prepared. Each scenario was prepared in two versions, one using UML-AD and the other, UML-ADE. Additionally, five defects were seeded into each diagram and two group of participants were asked to detect these defects according to the description document of the scenarios.

### Research procedure

**Figure 2:**
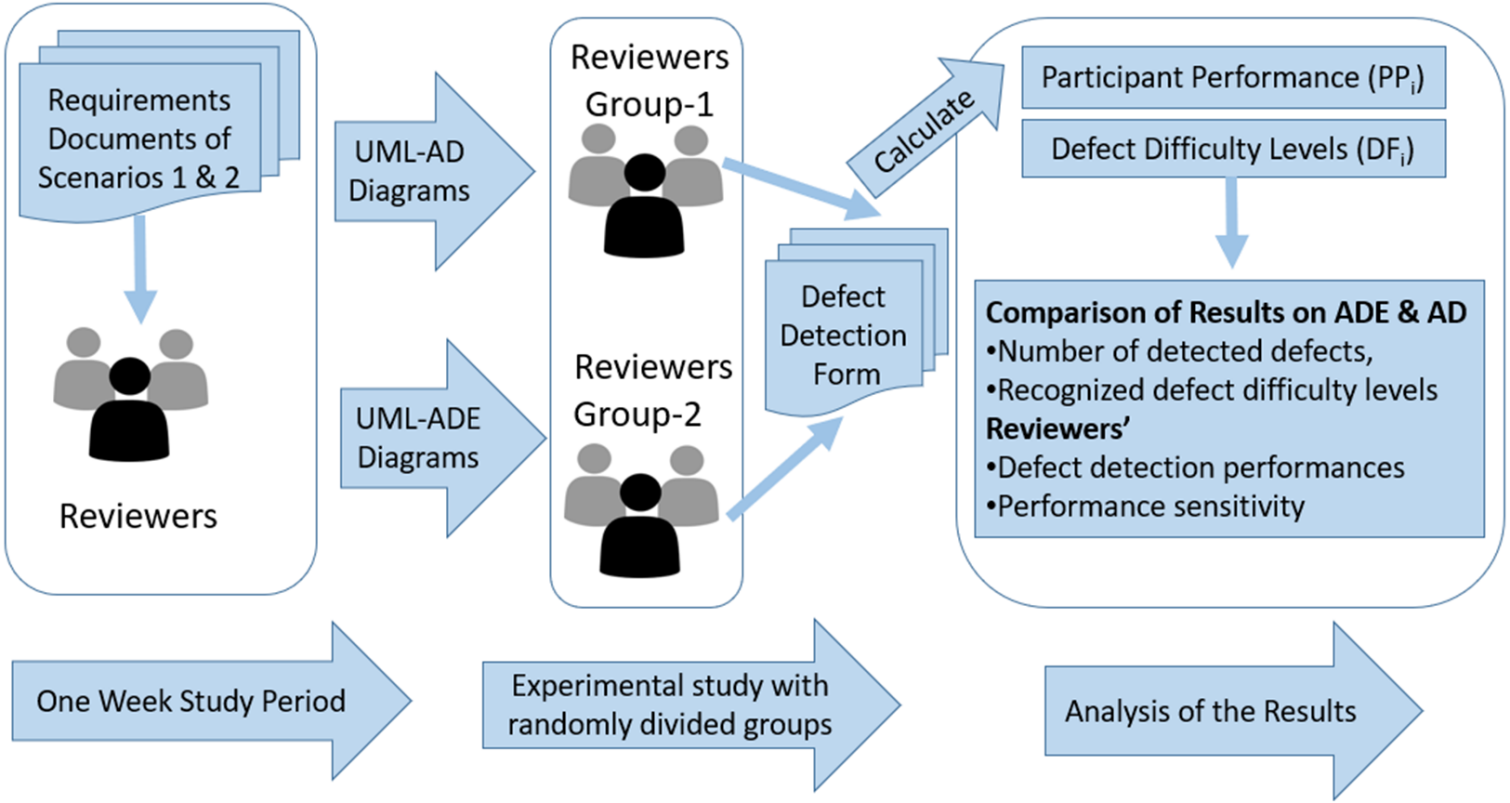
Research procedure of the study. Research method is explained.

This study was organized as a between-subjects empirical design. As explained in the Fig. 2, one week prior to the study, the participants were provided the user requirements documents prepared for each scenario. In this way, they had the opportunity to examine the contents and understand the main system requirements.

**Figure 3:**
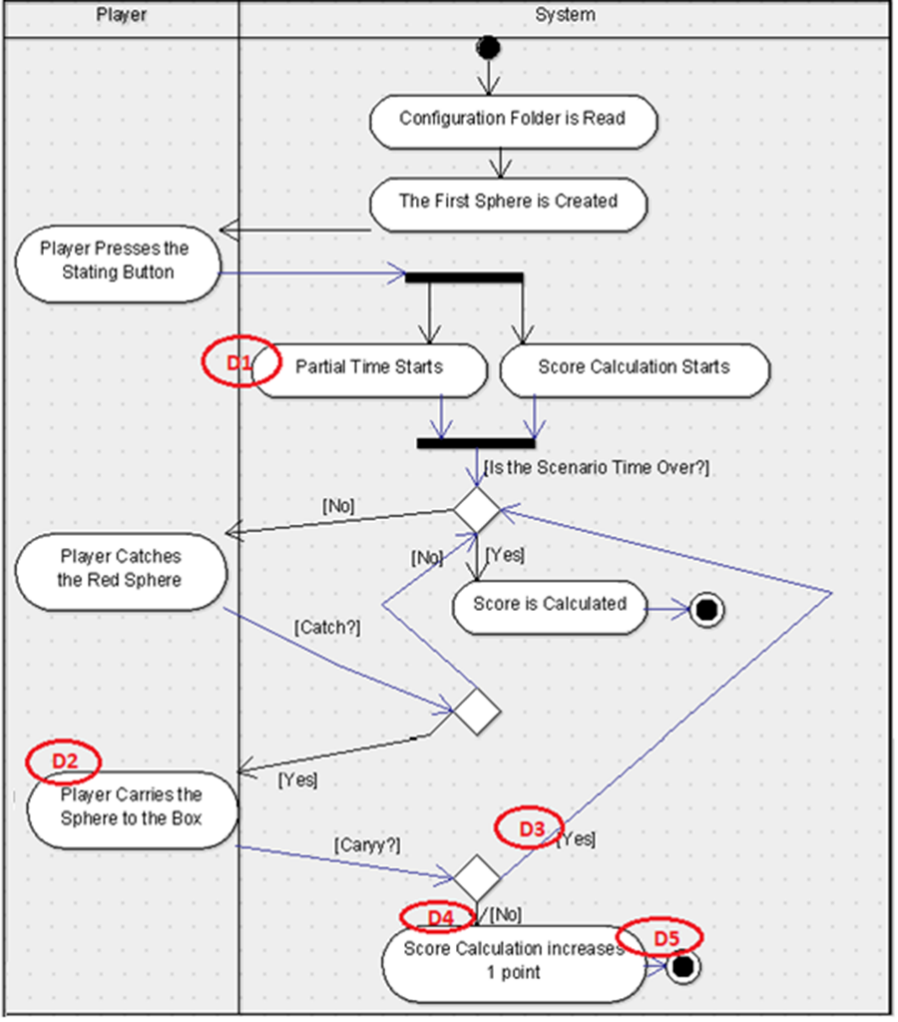
UML-AD model with defects for Scenario-1. Figure shows the defect distribution in Scenario 1 using UML-AD.

**Figure 4:**
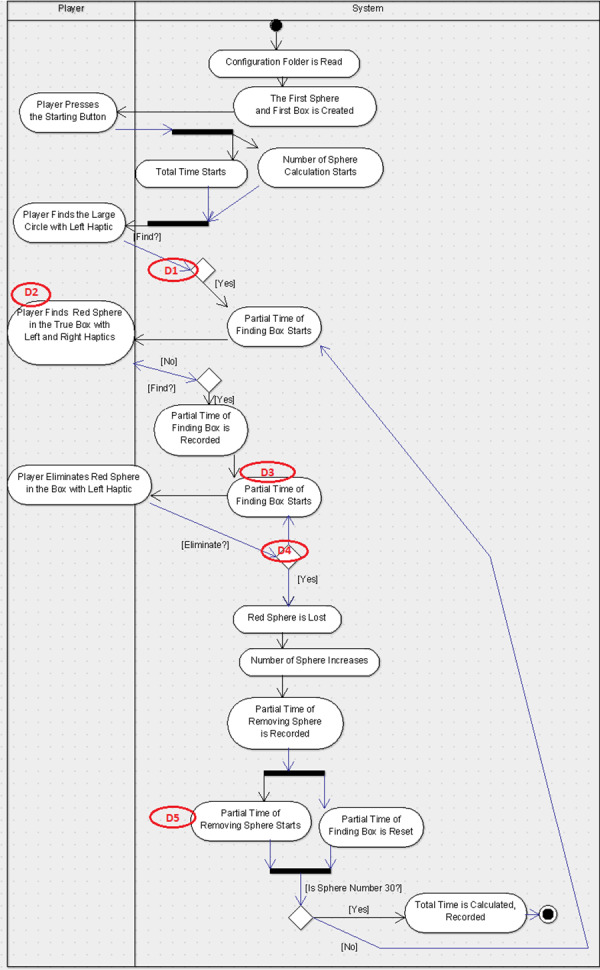
UML-AD model with defects for Scenario-2. Figure shows the defect distribution in Scenario 2 using UML-AD.

For the experimental study, the participants were randomly divided into two groups. The first group received the UML-AD representations for scenario-1 (See Fig. 3) and scenario-2 (See Fig. 4) which included five defects seeded-in, and the explanation document for UML-AD notation.

**Figure 5:**
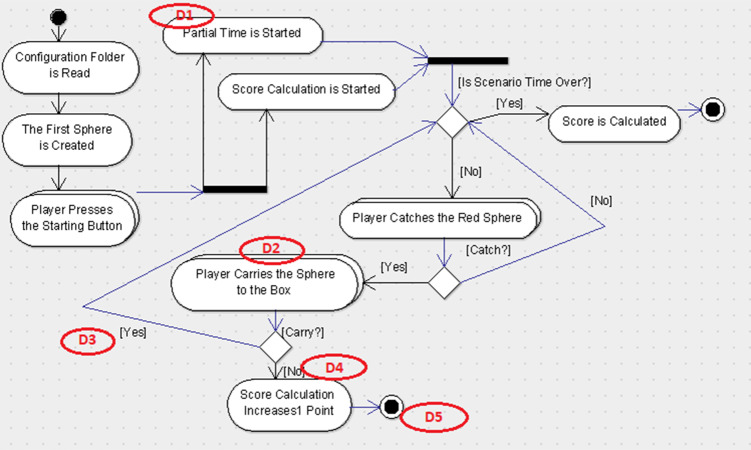
UML-ADE model with defects for Scenario-1. Figure shows the defect distribution in Scenario1 using UML-ADE.

**Figure 6:**
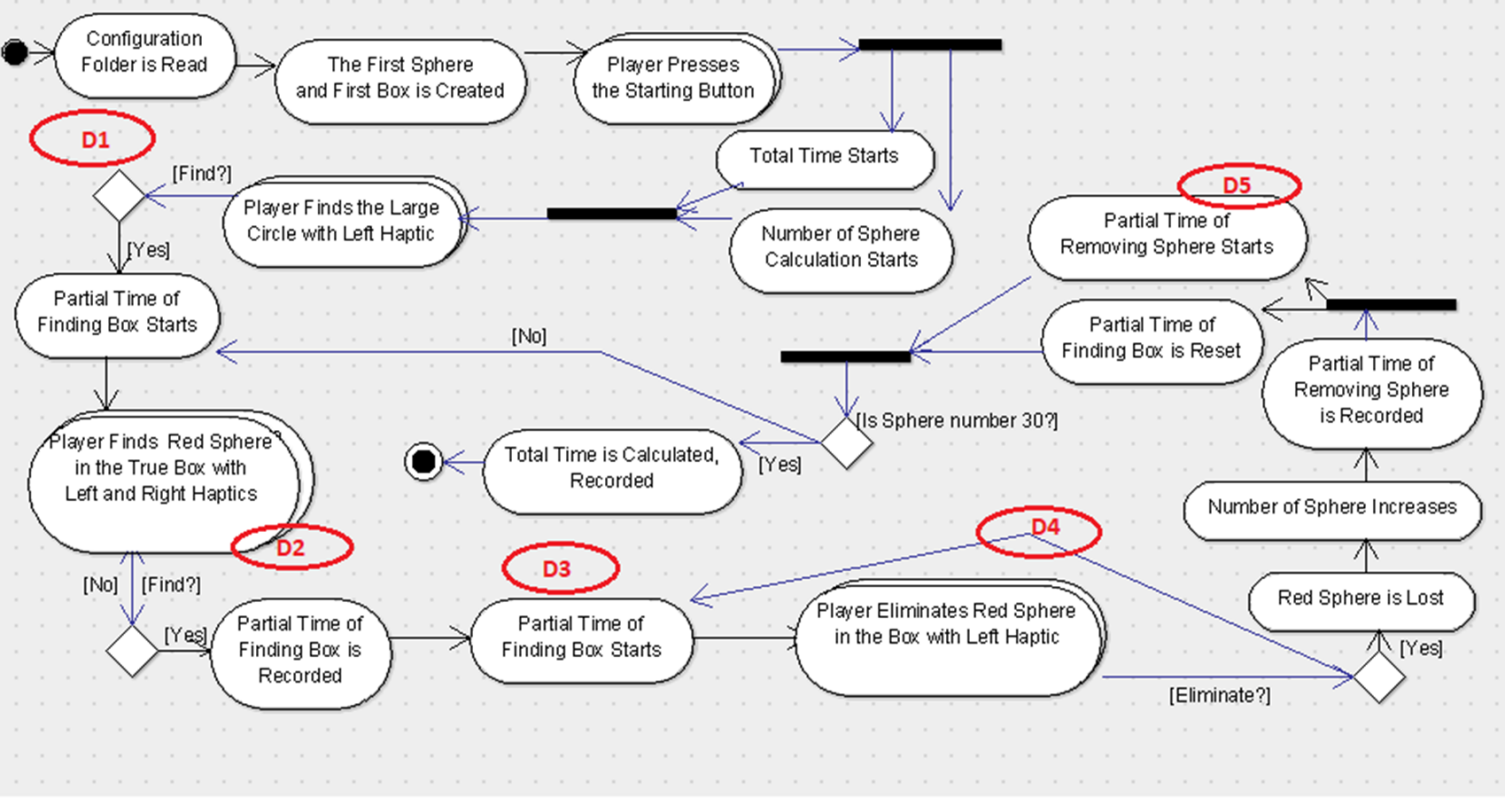
UML-ADE model with defects for Scenario-2. Figure shows the defect distribution in Scenario 2 using UML-ADE.

Similarly, the second group was provided the UML-ADE representations for scenario-1 (See Fig. 5) and scenario-2 (See Fig. 6) with the same five defects embedded in them and the explanation document for UML-ADE notation. During the experimental study with randomly divided groups, participants were asked to examine the UML diagrams according to the requirements document and to find the defects. They were informed about the number of defects and were given 50 min. to find them. Once the defects were found, the subject recorded them on the Web-based Defect Report Form. In order to better measure and compare each participant’s performance at defect detection, PP_*i*_ (Formula 2) and the difficulty levels of the defects DF_*i*_ (Formula 1) values were also calculated [30]. For the purpose of answering the research questions proposed in this paper, the data collected through the experimental study was analyzed both descriptively and statistically.

The research questions proposed in this study are:

RQ1: Can participants detect more defects in UML-ADE than in UML-AD?

RQ2: Are the defects seeded in UML-ADE easier to detect than in UML-AD?

RQ3: Do participants perform better on defect detection performance with UML-ADE than with UML-AD?

RQ4: Are the participants’ performance sensitivities higher with UML-ADE than with UML-AD?

### Instruments

The study is conducted in the participants’ native language, Turkish. Several instruments were prepared. These include the user requirements document of the scenarios, conceptual data model diagrams represented in the UML-AD and UML-ADE models for each scenario, Web-based Defects Report Form, questionnaires, notation explanation documents, and experimental study diagrams.

For this study, two scenarios were used which were developed for endoneurosurgery training programs (Cagiltay et al., 2019). Each scenario includes some user tasks intended to improve certain endoscopic surgical skills like depth perception, left–right hand coordination and eye-hand coordination in a 3D simulated environment. The users were asked to perform each task in a given time period. Besides the classical user interface, a gamification interface was applied to these scenarios. In this concern, the user score was shown in the interface with the parameters that were used for the calculation of the score for each task such as the duration of time spent on performing the task, the accuracy of the task (if it was performed successfully in a given time period or not), and the error rates indicating the number of wrong movements or unnecessary touches to the environment. Additionally, a sound feedback from the system was given for each successfully performed task. For each scenario, a user requirement document was prepared. The design diagrams of the scenarios were prepared with the notations UML-AD (Figs. 3 and 5, respectively) and UML-ADE (Figs. 4 and 6, respectively) representations and several defects as listed in Table 1 were seeded into these diagrams. In order to collect the necessary data to better understand the participants’ performance during the experimental study, a custom ‘Defect Report System’ software was developed. This web-based application recorded detailed information about the participant’s demographics, the scenario being studied, and the participants’ defect detection process. Each time the participant detects a defect, they were asked to record it in the Defect Report Form. In this way, measures such as the order in which the participant spotted a defect and the time it took to find defects were recorded (for each detected defect of two scenarios).

**Table 1:** Conceptual model defects seeded into scenarios. Descriptions for the defects seeded in the models.

**Code**	**Type**	**Description**
**Scenario-1**		
D1	Wrong Action State	Written action state is wrong. In this scenario, there is no partial time.
D2	Wrong Action State	Written action state is wrong. In this scenario, the user must drag away the sphere to the outside.
D3	Wrong Transition	If the user does not drag away the sphere to the outside, the total time must be controlled.
D4	Wrong Transition	If the user drags the sphere to the box, the reviewer receives a point.
D5	Irrelevant Final state	There cannot be a final state. Scenario time should be controlled.
**Scenario-2**		
D1	Missing Transition	Transition arrow must not be null. If there is a transition arrow, the condition must be defined. It also must in the “No” mode.
D2	Wrong Action State	The user must not remove the sphere with a left haptic. The user must remove the sphere with a right haptic.
D3	Wrong Action State	Besides the partial time of the box to be found, there must be a partial time for removing sphere.
D4	Missing Transition	If there is a transition arrow, condition must be defined. It also must be in “No” mode.
D5	Wrong Action State	The partial time for the sphere to be removed must be reset.

Finally, a questionnaire was also prepared and applied to better understand the participants’ feedback and opinions on the diagrams. As the participants were not expected to be familiar with the UML-AD notation, the explanations for both versions of these notations were also provided to the participants. Therefore, two notation explanation documents were also prepared. The material used for the experiment can be accessed from Supplemental Information.

### Participants

Seventy-two volunteers participated in the experimental study. These participants were senior-year students at the Departments of Computer Engineering, Software Engineering and Information Systems Engineering. Information regarding gender and the number of participants is given in Table 2. The participants were briefed about the procedure and their consent was obtained before the experiment.

**Table 2:** Participants. Number of Participants in each scenario.

	**Scenario**		
	** I**	** II**	**Total**
Female	8	9	17
Male	27	28	55
Total	35	37	72

Since the scenarios were distributed randomly to the participants, there is an uneven number of participants in Scenario-1 and Scenario-2 for both males and females. Accordingly, for Scenario-1 there were 35 participants, and for Scenario-2 there were 37 participants.

### Experimental study diagrams

For the experimental study, five defects for each scenario were seeded in both UML-AD and UML-ADE representations. The originals of these diagrams are in Turkish; for clarity, the English versions are provided below. The lists of the defects were seeded in each scenario as given in the Table 1.

For the experimental study, these defects were seeded in the UML-AD and UML-ADE versions of each scenario (See Figs. 3–6 respectively). As shown in the figures, Scenario-2 is more complex compared to Scenario-1.

### Measures

To analyze the defect detection experiment results, two measures proposed by Cagiltay et al. (2013) were used: Defect Detection Difficulty Level (DF) and Defect Detection Performance of a participant (PP). These measures are summarized below.

(1) DEFECT DIFFICULTY LEVEL (DF) MEASURE

In this measure, the DF value is calculated based on the average duration spent by all participants to detect the specified defect (*D*_*j*_), the score gained from the defect detection order (*O*_*j*_), Success rate of detecting defect *j* (*S*_*j*_- Number of people who detected defect *j*/Total number of participants) and the number of people who detected the specified defect (*D*_*j*_) (Cagiltay et al., 2013).

(1) \begin{eqnarray*}D{F}_{j}= \frac{{D}_{j}\bullet {O}_{j}}{{S}_{j}} .\end{eqnarray*}

(2) PARTICIPANT PERFORMANCE (PP) MEASURE

In this measure, the defect detection performance of each participant *i* (*PP*_*i*_) is calculated. To do so, the defect detection difficulty level value of each defect (*DF*_*j*_) value is used where *n* is the total number of defects detected by participant *i*, and *s* is the total number of defects seeded in the UML-AD &UML-ADE (Cagiltay et al., 2013).

(2) \begin{eqnarray*}P{P}_{i}= \frac{\sum _{j=1}^{n}D{F}_{j}}{\sum _{k=1}^{s}D{F}_{k}} .\end{eqnarray*}

This study is conducted as a part of Endoscopic Surgery Education (ECE) Project which is supported by The Scientific and Technological Research Council of Turkey (TUBITAK). The project number is 112K287 and Ethics Committee Report number is B.30.2.ATL.00.04.14/12-016. As the ethical committee approval is mandatory for TUBITAK, Atilim University Human Research Ethical Committee approved documents were submitted to TUBITAK. The ethical committee report covers informed consent form, and the samples of the data collection tools.

## Results

In this section, the results obtained are analyzed for each research question.

### Results on number of detected defects (RQ1)

**Figure 7:**
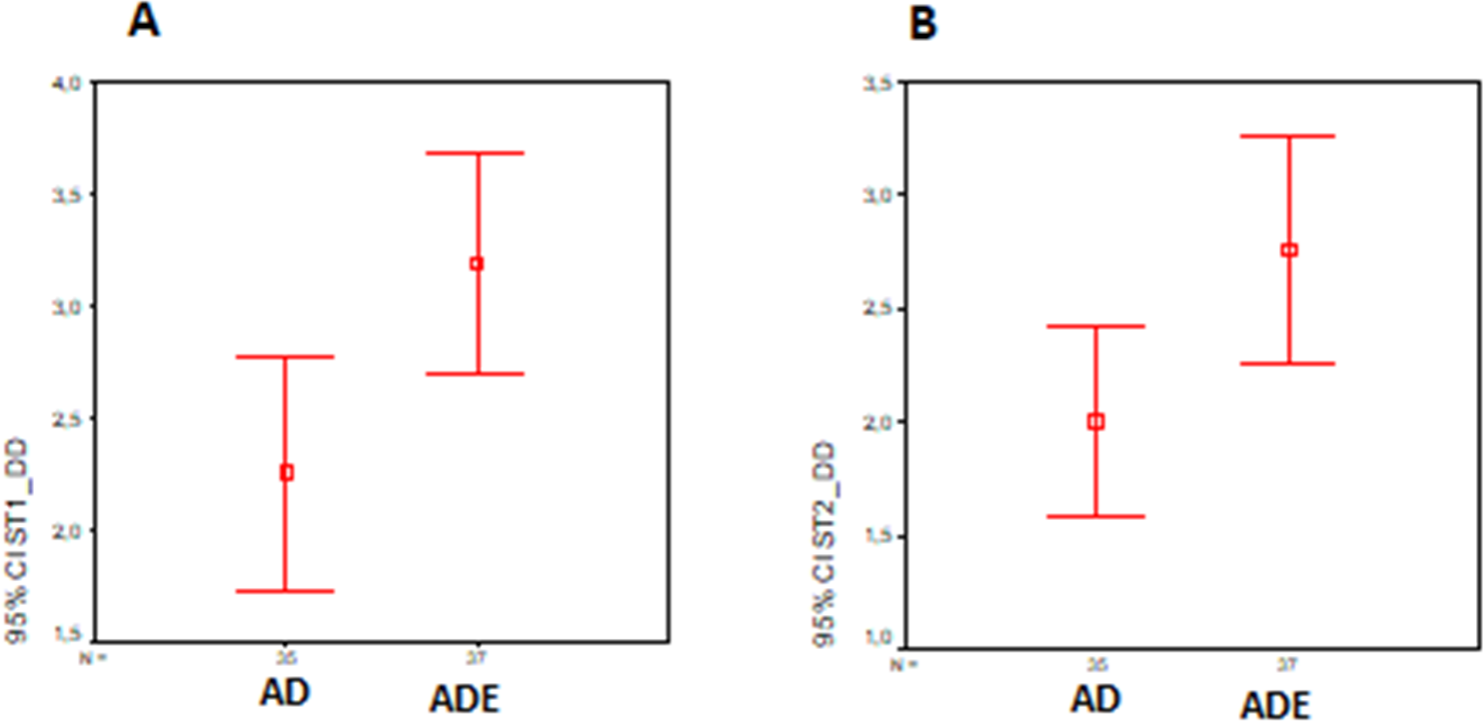
Detected defects distribution (A) Scenario-1. (B) Scenario-2. The figure shows the detected defect distribution in the experiment.

In order to answer RQ1, the number of defects detected by each participant was analyzed. Hence, an independent sample *t*-test was conducted to evaluate the hypothesis. It was found that those who worked on UML-ADE representation of Scenario-1 detected more defects than those working on UML-AD representation of the same scenario. The test was significant, *t*(70) = 2.63 and *p* = 0.011. The group working on UML-ADE detected more defects (*M* = 3.19, SD = 1.49) compared to the other group working with UML-AD ( *M* = 2.26, SD = 1.52). Figure 7A shows the distribution of both groups.

Similarly, an independent sample *t*-test was conducted to evaluate the hypothesis that the participants who worked on the UML-ADE representation of Scenario-2 detected more defects than those who worked on the UML-AD representation of the same scenario. The test was significant, *t*(70) = 2.35 and *p* = 0.022. Participants working on UML-ADE (*M* = 2.77, SD = 1.49) detected more defects than those working on UML-AD (*M* = 2.00, SD = 1.21). Figure 7B shows the distribution of both groups.

**Table 3:** Defect difficulty levels. The calculated defect difficulty levels for each scenario.

**Defect code**	**Scenario-1**		**Scenario-2**	
	**AD**	**ADE**	**AD**	**ADE**
D1	2,812	1,221	733	439
D2	1,728	1,313	3,049	1,728
D3	1,085	768	1,836	968
D4	1,647	941	1,202	1,374
D5	1,922	841	6,880	2,998
**Average**	1,839	1,017	2,740	1,501

### Results for recognized difficulty levels of the defects (RQ2)

For the second research question, the difficulty levels of each defect was calculated by *DFj* [30]. Table 3 illustrates the values of these calculated defect difficulty levels for Scenarios 1 and 2. It should be noted that all recognized difficulty levels for both scenarios are lower in the UML-ADE version than in the UML-AD except for the Scenario-2 in defect 4 (D4). Even these values are close (see Table 3, 1,374 and 1,202 respectively) compared to the differences in other defects on both scenarios; further analysis is required to determine the cause of this situation. However, as seen in Table 3, the average recognized difficulty level value for the defects seeded in the UML-AD of Scenario-1 (1,839) is higher than that of UML-ADE (1,017). Similar averages are achieved for the Scenario-2 on UML-AD (2,740) and on UML-ADE (1,501). These results indicate that it was more difficult to find the same seeded defects in the UML-AD version than in the UML-ADE version. In the same way, the defects seeded in UML-ADE (average 1,501) were identified more easily when compared to the ones seeded in UML-AD version (average 2,740). Because of the limited number of defects, a statistical analysis was not conducted for this data. The results of the questionnaire data also supported this result. The averages of participants’ responses for the three items of the questionnaire are summarized in Table 4, according to which the participants found the UML-ADE representations easier to understand (3.79) than UML-AD (3.07).

**Table 4:** Questionnaire responses. Questionnaire Responses for each notation.

**Questionnaire item**	**Notation**	
	** UML-ADE**	** UML-AD**
“I think ........ the diagram given to us is easy to understand.”	3.79	3.07
“How complicated is the given ........ the diagram, evaluate the complexity of the given diagram from 1 to 5. (1: very easy, 5: very difficult)	2.47	2.77
“I think I understand the system very well by looking at the given ........ diagram.”	3.79	3.37

Similarly, their estimates on the level of understanding of the game that is represented with the UML-ADE notations is higher (3.79) than that of UML-AD (3.37). Their responses likewise confirmed the complexity of the representations in UML-AD to be higher (2.77) than that of UML-ADE (2.47) (Table 4).

### Results about participants’ defect detection performance (RQ3)

**Figure 8:**
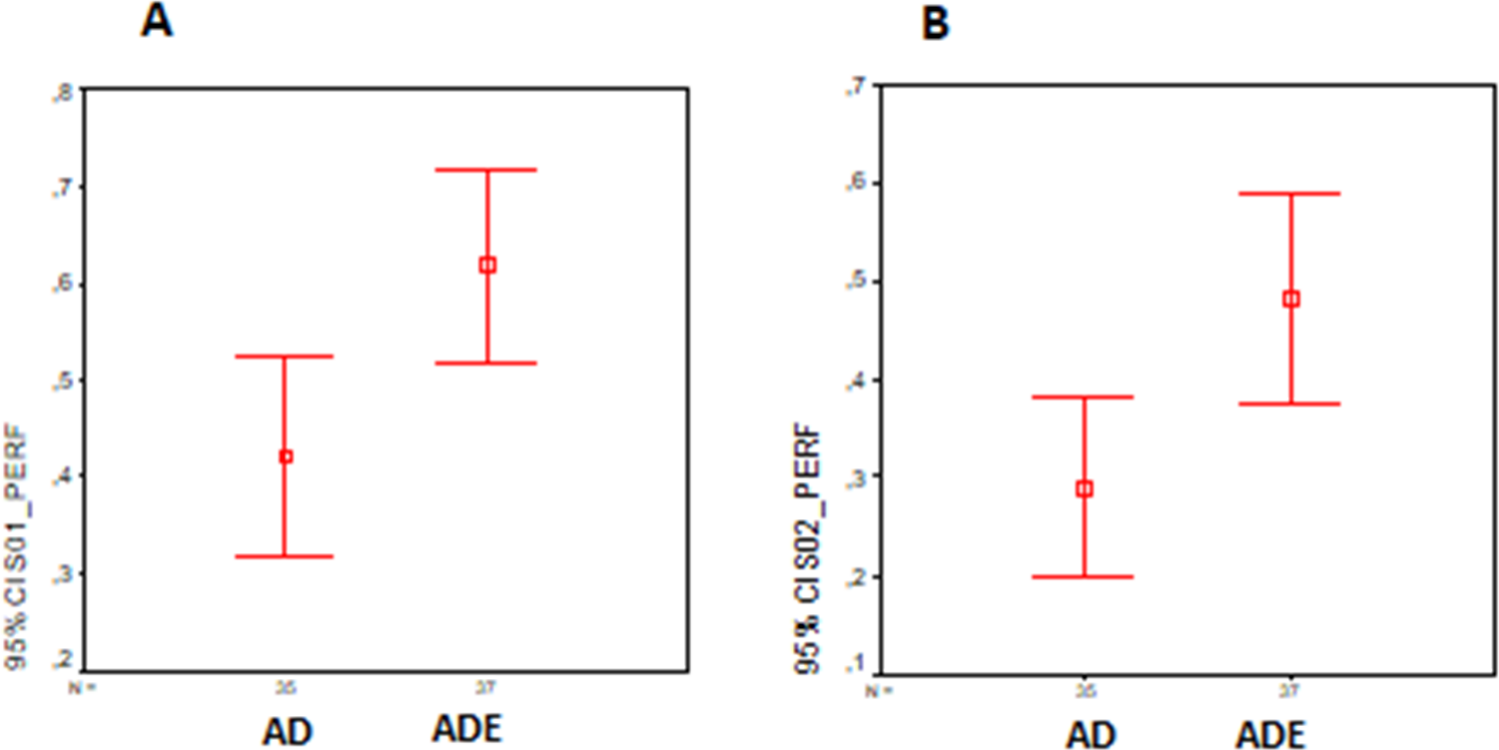
Defect detection performance distribution (A) Scenario-1. (B) Scenario-2. The figure shows the participants’ defect detection performance in the experiment.

Each participant’s defect detection performance (*PP*_*j*_) was calculated [30] to analyze the performance of participants of two groups. An independent sample *t*-test was conducted to evaluate the hypothesis that the participants’ performance in detecting defects on UML-ADE version is better than that on the UML-AD version for Scenario-1. The test was significant, *t*(70) = 2.77, *p* = 0.007. Participants working on UML-ADE performed better (*M* = .62, SD = .30) than the participants working on UML-AD (*M* = .42, SD = .30). Figure 8A shows the distribution of both groups.

Similarly, an independent sample *t*-test was conducted to evaluate the hypothesis that the participants’ performance in defect detection on UML-ADE is higher than it is on UML-AD for Scenario-2. The test was significant, *t*(70) = 2.76, *p* = 0.007. It was found that the participants working on UML-ADE performed better (*M* = .49, SD = .32) than those working on UML-AD (*M* = .29, SD = .27). Figure 8B shows the distribution of both groups.

### Results about Reviewers’ performance sensitivity (RQ4)

The True Positive (TP) and False Negative (FN) values for the detected defects were also calculated to better understand the performance of the participants. TP indicates the correctly-identified defects by each participant while the FN value indicates the incorrectly-rejected defects by each participant. Accordingly, the true positive rate, which is also called the ‘Sensitivity Measure’, which indicates how well a participant can detect a defect, is also calculated using the following formula (Peng, Wang & Wang, 2012).

(3) \begin{eqnarray*}Sensitivity= \frac{TP}{TP+FN} \end{eqnarray*}

An independent sample *t*-test was conducted to evaluate the hypothesis that the sensitivity value for the participants working on the UML-ADE version of the system design is higher than for the participants who worked on the UML-AD version. The test was significant, *t*(42) = 2.26, *p* = 0.26. The sensitivity value for the participants who worked on the UML-ADE version (*M* = 0.69, SD = 0.30), on average, is higher than that of the participants who worked on the UML-AD version (*M* = 0.57, SD = 0.33). In other words, the probability of the defect detection rate of the participants working on the UML-ADE version is significantly higher than that of the ones working on UML-AD.

## Discussion

This study hypothesizes that identification and separation of user tasks and computer system tasks improves the level of understandability of the software design. Accordingly, in this study, the importance of tasks during the software design process that identifies and differentiates between the user tasks and the computer system is highlighted.

In general, the findings of this study are summarized in Table 5, where the mean score of the number of detected defects for Scenario-1 (2.26 for UML-AD, 3.19 for UML-ADE) is higher than that of Scenario-2 (2.00 for UML-AD, 2.77 for UML-ADE).

**Table 5:** Summary of the results. Results of the experimental study is depicted.

**Notation**	**Scenario**		
		** I**	**II**
Number of detected defects	AD	2.26	2.00
	ADE	3.19	2.77
Defect detection performance	AD	0.42	0.29
	ADE	0.62	0.49
Recognized defect difficulty levels	AD	1,839	2,740
	ADE	1,017	1,501

It can be also observed that the recognized defect difficulty levels for Scenario-2 (UML-AD: 2,740, UML-ADE: 1,501) are higher than those of Scenario-1 (UML-AD: 1,839, UML-ADE: 1,017) for both UML representations. Parallel to this result, the recognized difficulty level of the defects in Scenario-2 (2,740 for UML-AD, 1,501 for UML-ADE) is higher than that of Scenario-1 (1,839 for UML-AD, 1,501 for UML-ADE). As reported earlier, this is because Scenario-2 was designed to be more complicated than Scenario-1. Hence, as expected, as the scenario becomes more complicated, the recognized defect difficulty level values increase and the participants’ performance drops, which can also be considered an indicator for validating the results of the study.

Another important result of this study is that the performance for the UML-ADE representations of both scenarios is higher than that of UML-AD representations of both scenarios, indicating that UML-ADE notation improves the understandability level of the representation. All these results show that by identifying the user tasks explicitly and representing them in the design documents in an integrated manner, UML-ADE representation potentially improves the software design quality.

According to our research questions, findings can be summarized as follows:

• Participants working on the UML-ADE model can detect more defects; therefore, understandability of UML-ADE is better with respect to UML-AD.
• The difficulty level of identifying defects in UML-ADE is lower than that of UML-AD.
• The participants’ performance in Scenario-1 is higher than in Scenario-2.
• Participants’ sensitivity values for those who work on the UML-ADE version is higher than the values for those who work on UML-AD version.

To use UML-ADE notation for a given system, designers need to identify user tasks and system tasks according to the system requirements provided. For the user tasks, double circles and for system tasks, a single circle (as shown in Fig. 1) could be used to represent and distinguish these two types of tasks. These tasks can be connected through flows as described in the requirements, which explicitly show relationship of each activity clearly. This approach represents different user tasks as an integrated manner, whereas swimlanes are employed to show separation of different user tasks for each user in the original UML-AD. This allows the separation of tasks into distinct swimlanes, creating an extra cognitive load which may cause the user’s attention to be split (Cierniak, Scheiter & Gerjets, 2009) and hence lower the comprehension of the software design.

## Conclusions

As a result of this study, it can be concluded that, for the design purposes of a software systems in general, and for serious game software specifically, when the proposed UML-ADE representations are used, defect detection performance and hence system quality can be improved. It can further be concluded that the understandability level of the UML-ADE notation is higher for representing the task flow in system design. In this way, the early detection of defects during the design phase of software development, in turn, improves the quality of the software product and reduces development costs. Besides, the results of this study indicate that the identification of the user task in the design document has a potential to improve the quality of the software design. This may improve software testing and maintenance processes, the functionality, feedback mechanisms, user-experience and human–computer interaction capabilities of the systems, all of which to be further researched.

These results encourage researchers to develop specific design representations dedicated to task design. Additionally, the potential impact of specific design representations on different software products, where identification of user task has an importance, also needs to be further researched. Finally, additional solutions to identify and differentiate the tasks of the users with different roles in the software systems may also be explored.

##  Supplemental Information

10.7717/peerj-cs.503/supp-1 Supplemental Information 1 Questionnaire about UML-ADE design

10.7717/peerj-cs.503/supp-2 Supplemental Information 2 Questionnaire about UML-AD design

10.7717/peerj-cs.503/supp-3 Supplemental Information 3 Questionnaire responses

10.7717/peerj-cs.503/supp-4 Supplemental Information 4 Dataset 1

10.7717/peerj-cs.503/supp-5 Supplemental Information 5 Supplemental Material Scenarios and notation explanation

## Additional Information and Declarations

### Data Availability

The following information was supplied regarding data availability: Raw measurements and calculations performed for the experiment are available in the Supplementary Files
