# Supplementary material for: A user task design notation for improved software design
Source: PeerJ Comput Sci. 2021 May 24;7:e503. doi: 10.7717/peerj-cs.503 (PMC8156997; doi:10.7717/peerj-cs.503)
Supplement: Supplemental Information 1 [file peerj-cs-07-503-s001.pdf]

QUESTIONNAIRE ABOUT UML-ADE DESIGN

Some expressions about UML-ADE diagram that you currently worked on are given below. About these expressions choose one of the five alternatives (from 1 to 5).

		1 STRONGLY NOT AGREE	2 NOT AGREE	3 DON' T KNOW	4 AGREE	5 STRONGLY AGREE
1.	I think UML-ADE diagram given to us is easy to understand.	☐	☐	☐	☐	☐
2.	How complicated is the given UML-ADE diagram, evaluate from 1 to 5 (1: very easy - 5: very difficult)	☐	☐	☐	☐	☐
3.	I think I understand the system very well by looking to the given UML-ADE.	☐	☐	☐	☐	☐

4. What is your opinion or suggesstions about UML-ADE design or intelligibility?

____
____
____
____

5. If you design the Collection Spheres in the Room game (1. Scenario), how can you design? Draw.

6. If you design the Catching Spheres in the Box game (2. Scenario), how can you design? Draw.
