# Supplementary material for: A user task design notation for improved software design
Source: PeerJ Comput Sci. 2021 May 24;7:e503. doi: 10.7717/peerj-cs.503 (PMC8156997; doi:10.7717/peerj-cs.503)
Supplement: Supplemental Information 5 — Scenarios and notation explanation [file peerj-cs-07-503-s005.zip › Supplementary Material_UML_AD_ADE/Notation Explanation_UML_AD.pdf]

UML-AD EXPLANATION DOCUMENT

UML-AD diagrams are drawn with using ArgoUML tool that is presented to you. In these diagrams, various control flows are used. Control flows mean the flow of a model. According to UML-AD control flows, you should analyze these diagrams.

UML-AD Control Flows:

1. (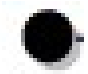) **Initial Node:** This is initial point. Activity starts with initial point.
2. (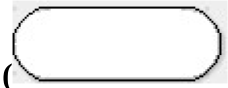) **Action:** This node is used to define the all actions. There is no showing message when from an activity to another activity flow.
3. (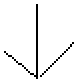) **Control Flow:** This node supplies connection between action. Control action is used to pass from one action to another action.
4. (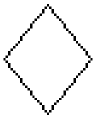) **Decision Node:** This node is used to define a condition in an activity. There is a one incoming flow and multiple outgoing flows. There is a situation that is depended on every outgoing flow and to explain this situation, brackets are used.
5. (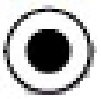) **Activity Final Node:** This node is final point. When the activity finishes, final point is reached. There can be more than one final node.
6. (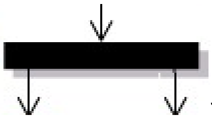) **Fork Node:** One incoming flow is separated to more than one outgoing flow that is simultaneous.

(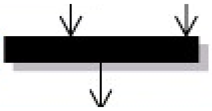 **) Join Node:** More than one incoming flow can combine with one outgoing flow that is simultaneous. This node defines to combine the parallel actions and continue with a one action.

Available Defects: During the design of UML diagrams, for the scenario that is given for you, there are some defects about situations that is shown in below.

- Wrong Transition
- Missing Transition
- Irrelevant Finite State
- Wrong Action State
- Missing Final State

It is expected for you to find defects with marking them on diagrams and save them to the system. You should press "Submit Defect" button after you save every defect to the system. So, time of finding defects is shown in the screen. When the finding defect process is completed, you should press the "Submit All" button and complete this study.
