# Supplementary material for: A user task design notation for improved software design
Source: PeerJ Comput Sci. 2021 May 24;7:e503. doi: 10.7717/peerj-cs.503 (PMC8156997; doi:10.7717/peerj-cs.503)
Supplement: Supplemental Information 5 — Scenarios and notation explanation [file peerj-cs-07-503-s005.zip › Supplementary Material_UML_AD_ADE/Scenario1_Requirement Document.pdf]

USER REQUIREMENTS DOCUMENT FOR SCENARIO 1

This scenario passes in a room. Player acts backward and forward with using 1 haptic device in the room. Configuration folder is read by system. In this folder, there are sphere size, distance, wall shape, lines, sphere color frames. Also, the first sphere is created by system. Processes that are in the scenario starts within the player press the starting button. Player’s the main task is to collect red spheres from the different point of the room and carry them to the outside. In this scenario, scenario time and score calculation is defined. Scenario time is total times that all identified spheres are carried to the outside after they are caught. The score calculation increases with the player carries every sphere to the outside. Scenario time and score calculation starts when the scenario starts. Scenario time control is done. If the scenario time does not finish, player should catch every sphere in the room. If the player catches every sphere in the room, s/he should carry it to the outside. When the player carries the sphere to the outside, score calculation increases 1 point and scenario time is control again. If the total time is not over that is for scenario, scenario processes continues repeatedly with returning the beginning. If the total time is over that is for the scenario, total score is calculated for the player catches the sphere and carries it to the outside and the scenario finishes.
