# Supplementary material for: A user task design notation for improved software design
Source: PeerJ Comput Sci. 2021 May 24;7:e503. doi: 10.7717/peerj-cs.503 (PMC8156997; doi:10.7717/peerj-cs.503)
Supplement: Supplemental Information 5 — Scenarios and notation explanation [file peerj-cs-07-503-s005.zip › Supplementary Material_UML_AD_ADE/Scenario2_Requirement Document.pdf]

USER REQUIREMENTS DOCUMENT FOR SCENARIO 2

This scenario passes in a room. Player acts backward and forward with using 2 haptic devices in the room. Configuration folder is read by system. In this folder, there are sphere size, distance, wall shape, lines, sphere color frames. Also, the first sphere and first box are created by system. Processes that are in the scenario starts within the player press the starting button. Player's the main task is to find red spheres from the boxes that have different angles with left or right haptics and eliminate them.

In the scenario, when the game starts, total time and number of sphere calculation also starts. Total time continues until the player success to eliminate the 30 spheres from the boxes that have different angles. Player should find the large circle with left haptic. This large circle helps player to eliminates the sphere with right haptic. If the player does not find the large circle, this process is repeated until the circle is found. If the player finds the large circle, partial time of finding box and player should find the spheres from the boxes that have different angles. When the player does not find process continues until sphere is found. If the player finds the spheres from the boxes that have different angles, partial time of finding box is recorded and partial time of removing sphere starts. Partial time of removing sphere is time that the player eliminates the red sphere with the right haptic in the box. When the sphere is eliminated each, partial time of removing the sphere is reset. Player should eliminate the red sphere in the true box with right haptic. If the player eliminates the red sphere, the red sphere is lost; the number of sphere increases, partial time of removing the sphere is recorded and reset, partial time of finding box is reset, and until the number of sphere is 30, the red sphere occurs another box. If the player does not eliminate the red sphere, the sphere cannot be lost, partial time of removing sphere starts and player eliminates the red sphere in the true box with right haptic. When the player successes to eliminate the 30 spheres, total time are recorded, and scenario is completed. If the player does not success to eliminate the 30 spheres, scenario returns the 'partial time of finding box' process and scenario processes continues repeatedly.
