# Supplementary figures and images for: A user task design notation for improved software design
Source: PeerJ Comput Sci. 2021 May 24;7:e503. doi: 10.7717/peerj-cs.503 (PMC8156997; doi:10.7717/peerj-cs.503)

## UML-AD MODEL USED FOR SCENARIO 1

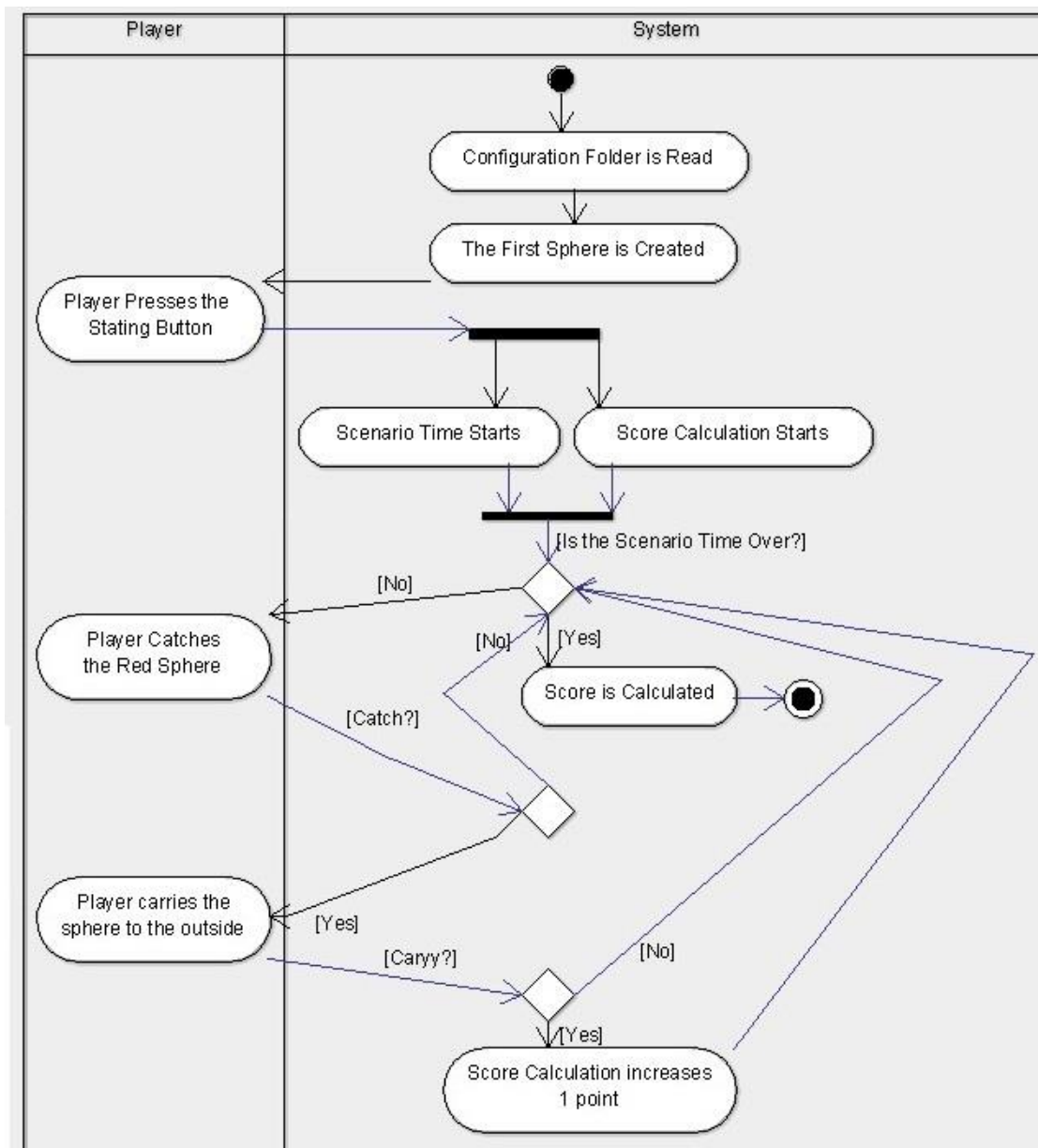

Supplement: Supplemental Information 5 — Scenarios and notation explanation [file peerj-cs-07-503-s005.zip › Supplementary Material_UML_AD_ADE/Scenario1_UML_AD.pdf]

## UML-ADE MODEL USED FOR SCENARIO 1

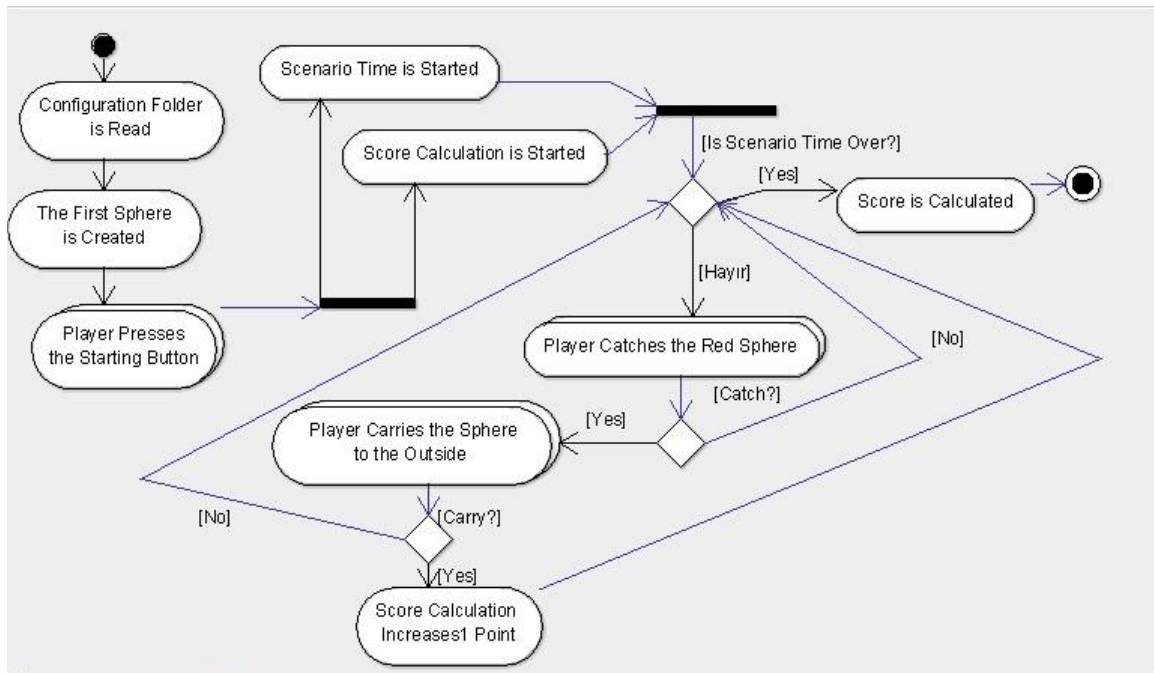

Supplement: Supplemental Information 5 — Scenarios and notation explanation [file peerj-cs-07-503-s005.zip › Supplementary Material_UML_AD_ADE/Scenario1_UML_ADE.pdf]

## UML-AD MODEL USED FOR SCENARIO 2

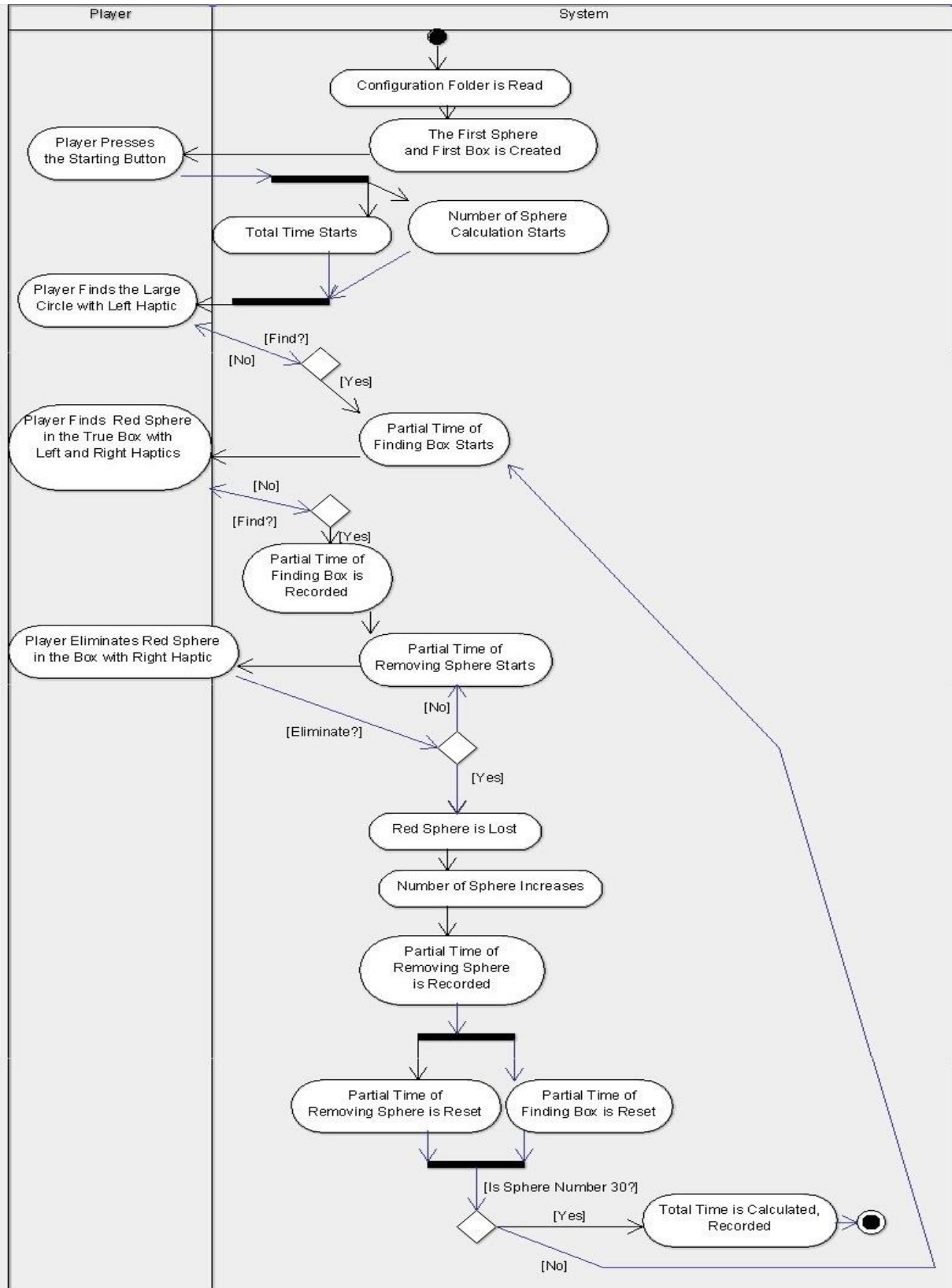

Supplement: Supplemental Information 5 — Scenarios and notation explanation [file peerj-cs-07-503-s005.zip › Supplementary Material_UML_AD_ADE/Scenario2_UML_AD.pdf]

## UML-ADE MODEL USED FOR SCENARIO 2

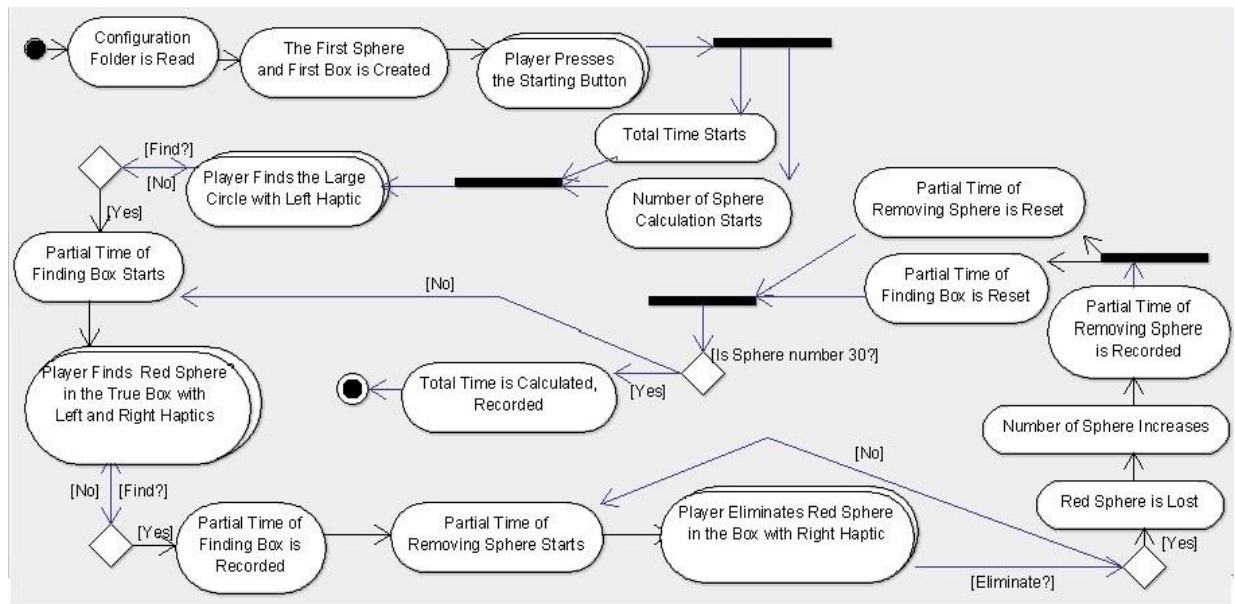

Supplement: Supplemental Information 5 — Scenarios and notation explanation [file peerj-cs-07-503-s005.zip › Supplementary Material_UML_AD_ADE/Scenario2_UML_ADE.pdf]
